# Determination of equilibrium dissociation constants for recombinant antibodies by high-throughput affinity electrophoresis

**DOI:** 10.1038/srep39774

**Published:** 2016-12-23

**Authors:** Yuchen Pan, Eric K. Sackmann, Karolina Wypisniak, Michael Hornsby, Sammy S. Datwani, Amy E. Herr

**Affiliations:** 1 University of California, Berkeley – UCSF Graduate Program in Bioengineering, Berkeley, CA, 94720, USA; 2 Labcyte Inc., 1190 Borregas Ave, Sunnyvale, CA, 94089, USA; 3 Department of Pharmaceutical Chemistry, University of California, San Francisco, CA, 94158 USA; 4 Department of Bioengineering, University of California, Berkeley, CA, 94720, USA

## Abstract

High-quality immunoreagents enhance the performance and reproducibility of immunoassays and, in turn, the quality of both biological and clinical measurements. High quality recombinant immunoreagents are generated using antibody-phage display. One metric of antibody quality – the binding affinity – is quantified through the dissociation constant (K_D_) of each recombinant antibody and the target antigen. To characterize the K_D_ of recombinant antibodies and target antigen, we introduce affinity electrophoretic mobility shift assays (EMSAs) in a high-throughput format suitable for small volume samples. A microfluidic card comprised of free-standing polyacrylamide gel (*fs*PAG) separation lanes supports 384 concurrent EMSAs in 30 s using a single power source. Sample is dispensed onto the microfluidic EMSA card by acoustic droplet ejection (ADE), which reduces EMSA variability compared to sample dispensing using manual or pin tools. The K_D_ for each of a six-member fragment antigen-binding fragment library is reported using ~25-fold less sample mass and ~5-fold less time than conventional heterogeneous assays. Given the form factor and performance of this micro- and mesofluidic workflow, we have developed a sample-sparing, high-throughput, solution-phase alternative for biomolecular affinity characterization.

Immunoreagents are notorious for variation in quality and performance^1^,^2^. Differences in specificity, binding affinity, and even lot-to-lot performance are widely reported, negatively impacting resources and reporting^3^. Consequently, integrated approaches for the generation and characterization of immunoreagents are needed^2^,^4^,^5^. Such developments would enhance the performance characteristics of recombinant antibody libraries (with sequence databases guiding molecular design)^2^, antibody phage display^6^, antibody yeast display^7^ and even virtual affinity maturation approaches^8^,^9^. Controlled generation of well-characterized antibodies would be a benefit to immunoreagents and immunotherapy^8^,^10^,^11^.

Yet, despite the importance of robust immunoreagents to fields spanning the biosciences to biomedicine^12^,^13^,^14^, no consensus exists on guidelines or standardized methods for determining antibody quality^15^. Specificity is an important consideration in antibody quality, as is binding affinity, which is quantified through the equilibrium dissociation constant, K_D_^16^,^17^. The K_D_ of a binding pair can be assessed using surface-based (heterogeneous) methods including surface plasmon resonance (SPR)^18^, biolayer interferometry (BLI)^19^ and enzyme linked immunosorbent assays (ELISA)^20^. While recent SPR instrument advances make the assay high-throughput, most K_D_ assays require hours per measurement, thus limiting relevance. And even high-throughput forms of SPR see copious reagent consumption, a limitation in some applications^21^. Moreover, all surface-based measurements suffer from mass transport limitations that increase the time for a reaction to reach equilibrium^22^. In fact, some reactions may never reach equilibrium, making these assays suitable for assessing relative binding only^23^,^24^. Heterogeneous assays are further confounded by non-specific surface absorption of proteins^25^,^26^.

Homogeneous assays, such as affinity capillary electrophoresis (ACE), are a solution-phase alternative for molecular binding. ACE does not suffer from complications related to surface immobilization^27^. ACE was pioneered in the 1960 s^28^ and is now applied to a wide variety of molecules and kinetic regimes. For details on both fundamental and applied aspects of ACE, we direct the reader to Winzor^29^. One type of ACE assay – the electrophoretic mobility shift assay (EMSA) – detects binding-induced changes in electrophoretic mobility (e.g., size, conformation and charge) with microfluidic design yielding precise and high-throughput versions^30^,^31^,^32^.

Until recently, custom microdevices and operational equipment^33^ limited suitability for high-throughput antibody K_D_ measurements (e.g., screening), but new low-infrastructure and high-throughput versions are emerging. Microchannel-free gel electrophoresis assays^34^,^35^,^36^,^37^ may provide a suitable alternative. Two examples of the format include the microplate gel array described by Gaunt *et al*.^34^ and the free-standing polyacrylamide gel electrophoresis (*fs*PAGE) array described previously by our own group^35^,^38^. Regarding the latter, the *fs*PAG design comprises a planar polyacrylamide gel (PAG) lane and sample reservoir fabricated by UV-based photopatterning. We have detailed *fs*PAG arrays for 96 and 384 simultaneous electrophoretic separations – one per lane – all located on one monolithic polymer ‘card’ and operated using a single power source and two electrodes. Most relevant to the present study is our previous optimization of 96 concurrent *fs*PAGE EMSAs to assess binding of the Vc2 riboswitch aptamer with increasing concentrations of its small molecule ligand, cyclic di-GMP^38^.

Here, we developed and applied the EMSA card assay to report antibody K_D_ values in a manner suitable for use in existing, automated recombinant antibody production workflows. Given the sample size, number of dilution points, and replicate samples, we automated sample dispensing onto the EMSA card using an automated acoustic droplet ejection (ADE) technology^39^. ADE is a non-contact, low-volume droplet delivery technology that enables fast repetition rate, positional accuracy, positional precision, volume accuracy, and precision liquid handling. Focused high-frequency sound waves create a controlled pressure wave front that can be further excited near the fluid surface to generate and eject small droplets. Due to advances in computational speed and signal processing algorithms, ADE precisely controls droplet generation to yield predefined volumes and ejection speeds^40^,^41^. We then characterized and applied the automated system to report K_D_ for a six-member library of recombinant antibodies against eGFP.

## Results

### Principle of K_D_ determination by affinity electrophoresis

Given the molecular binding reaction at equilibrium, A + B = AB, where A is the immunoreagent, B is the protein target, and AB is the immunocomplex. K_D_ is then defined as:


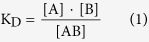


Here [A], [B], and [AB] are now the concentrations of immunoreagent, protein target, and immunocomplex, respectively. To determine K_D_ via EMSA, an electrophoretic immunoassay is performed on equilibrated samples having a fixed [A] and a range of [B] spanning from K_D_/10 to 10K_D_, which is a common experimental design space^42^. EMSAs report K_D_ by measuring the electrophoretic mobility difference (shift) between the bound and unbound forms of a target analyte A and immunocomplex AB. When the binding reaction places the EMSA in the slow interconverting kinetics regime, the EMSA measures the area-under-curve (AUC) for the immunocomplex (AB) and the immunoreagent peaks. When the reaction places the EMSA in a fast interconverting kinetics regime, the EMSA measures the mobility of each resulting band. With the [B] value known, the K_D_ is determined by least-squares regression^42^ to:


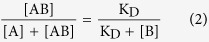


Here, the equation is expressed as the ratio of AB to total A, where total A is the sum of free A and bound A (in AB). K_D_ is thus sensitive to the accuracy of the AUC measurement or mobility measurement (depending on the kinetic regime). In this study, we considered the binding reaction between the enhanced green fluorescence protein (eGFP) and a pilot six-member library of Fab antibody fragments, generated by the antibody phage display pipeline of the Recombinant Antibody Network (RAN) at UCSF^43^.

We first considered the EMSA separation in light of both the physicochemistry and kinetics of the Fab fragment and eGFP binding reactions. An important factor in EMSA separation performance is the difference between the time scale of electromigration and rate of interconversion between the immunocomplex and the free protein. Depending on the kinetic regime of the binding reaction, one may observe either two distinct protein peaks (a slower eGFP-Fab immunocomplex peak and a faster free eGFP peak) or co-migration of the two species, yielding a single detectable band that migrates with the weighted average electrophoretic mobility of the species^33^.

To predict behavior for our EMSA separation, we defined a dissociation Damköhler number (Da_off_ = *k*_off_L/Eμ) to quantify the relative rates of the dissociation reaction to electromigration. Here, *k*_off_ is the kinetic dissociation rate (s^−1^), *L* is the separation length (mm), *E.* is the applied electric field strength (V/mm), and μ is the electrophoretic mobility of the molecule (mm^2^/Vs). In the kinetic regime where Da_off_ ≪ 1, the dissociation is much slower than electromigration, and the eGFP-Fab fragment immunocomplex and free eGFP protein separate from each other during electrophoresis. In contrast, in the regime where Da_off_ ≫1, the eGFP-Fab fragment immunocomplex and the free eGFP will either co-migrate as one band (fast association) or the immunocomplex band may dissociate/disperse (slow association).

**Figure 1:**
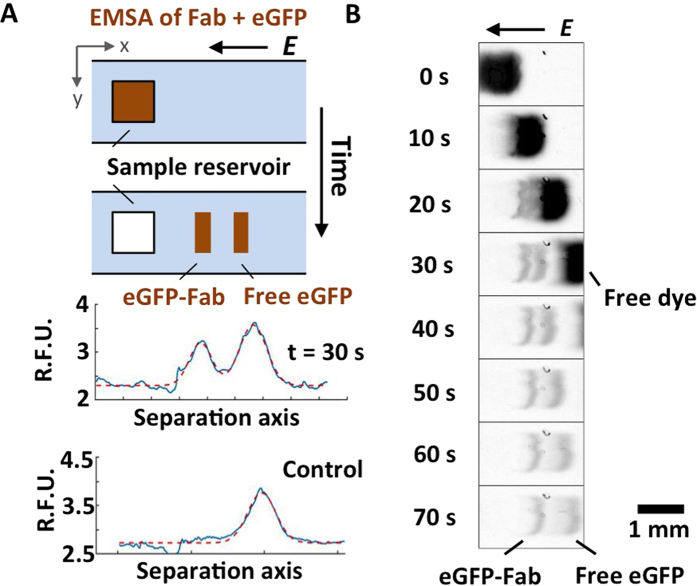
Principle and optimization of K_D_ determination by EMSA. (**A**) Schematics of EMSA separation of recombinant Fab fragment (Fab) and eGFP. By EMSA, the eGFP-Fab immunocomplex has a larger molecular mass and therefore lower electrophoretic mobility than unbound (free) eGFP. Fluorescence intensity profiles for EMSA-based analysis of eGFP and Fab reaction and a negative control (no Fab fragment) are shown (elapsed t = 30 s, E = 50 V/cm; [Fab] = 5 nM; [eGFP] = 10 nM). (**B**) Time evolution of inverted grayscale fluorescence micrographs show the eGFP-Fab immunocomplex peak resolved from free eGFP peak by EMSA. R.F.U. Relative fluorescence units.

To empirically determine the kinetic regime of the EMSA for this binding system, we estimated that each Fab will likely have 1 × 10^−4^ s^−1^< *k*_off_ <1 × 10^−3^ s^−1^ (data from Octet measurements; Figure S1), resulting in a Da_off_ ~0.05. Consequently, we anticipate two distinct and resolvable peaks by EMSA (Fig. 1A). Further, given the anticipated K_D_ of ~10^−9^ M, the EMSA should assess eGFP across a concentration range spanning 0.1 K_D_ <[eGFP] <10 K_D_. Consequently, to measure sub-nanomolar K_D_ values, we conjugated eGFP with the fluorophore AF647 to ensure sufficient detection sensitivity. On the EMSA card, each *fs*PAGE separation unit houses ~10^−2^ ng to ~10^−1^ ng of material (antigen concentration: ~10^−9^ M, *fs*PAGE, antigen molecular weight: 2 × 10^4^ Da and sample reservoir volume: 4 × 10^−6^ L; total mass: 10^−9^ × 2 × 10^4^ × 4 × 10^−6^ = 8 × 10^−10^ g) and, therefore, neither intrinsic eGFP fluorescence nor protein staining (lower detection limit: ~100 ng for classical Coomassie Blue staining and ~1 ng for silver staining)^44^ are sufficiently sensitive. While labeling of antigen with fluorophore may be prohibitively sample-consuming and/or expensive in certain applications, recombinant antibody screening utilizes a stock of a single target antigen. As multiple recombinant antibodies are tested against a single stock solution of target antigen, use of a fluorescently labeled target antigen is feasible. In the reverse case, when the immunoreagent is plentiful (i.e., immunoassays), direct antibody labeling is a common approach. Therefore while not universally appropriate, fluorophore conjugation of a target antigen stock is suitable for preliminary antibody screening prior to deeper characterization of each promising candidate immunoreagent. Using the single EMSA unit (*E* = 50 V/cm), we observed a low mobility eGFP-Fab immunocomplex peak, a higher mobility free eGFP peak (excess eGFP) and the fastest peak associated with unbound, excess AF647 fluorophores (Fig. 1B). The unbound fluorophore peak acts as an electromigration control. In a negative control with no Fab fragment present, we detected no eGFP-Fab immunocomplex peak (Fig. 1A).

To next optimize the appropriate separation duration for the 384-plex EMSA card, we monitored the separation resolution (*SR*) between the eGFP-Fab immunocomplex and the free eGFP peak (*SR* = ΔL/(0.5(4σ_1_ + 4σ_2_)), where ΔL is the peak-to-peak displacement and 4σ is the width of each Gaussian concentration profile^45^. Two peaks are considered resolved when *SR* ≥1.0. For E = 50 V/cm, the EMSA *SR* exceeded unity after just 30–35 seconds of elapsed separation time.

### Optimizing microfluidic EMSAs for high-throughput K_D_ determination

**Figure 2:**
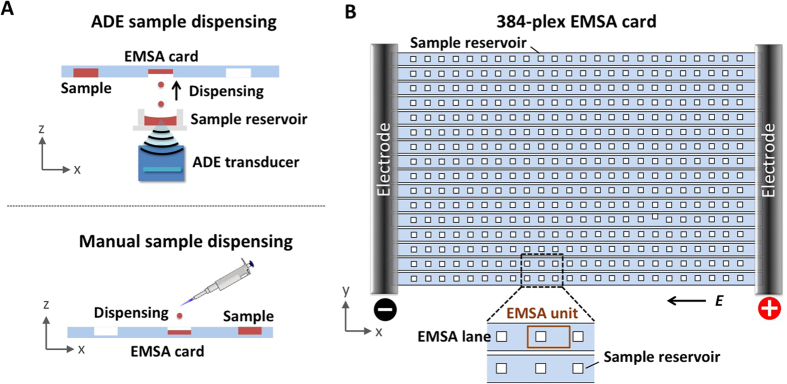
Schematic design of sample dispensing schemes and EMSA card for high-throughput K_D_ determination. (**A**) Side-view schematics of sample dispensing via manual pipetting or acoustic droplet ejection (ADE) to one of the 384 sample reservoirs on the EMSA card. (**B**) Top-view schematics of the 384-plex EMSA card design.

We next designed an *fs*PAG card to support 384 concurrent EMSAs in an “open” format (EMSA card) compatible with commercial liquid dispensing systems, including the ADE system studied here (Fig. 2A). Each of the 384 unique “EMSA units” is composed of a 1 × 1 mm^2^ sample reservoir and a contiguous electrophoretic separation lane that is 4 mm wide (Fig. 2B). The footprint of 384-plex EMSA card corresponds to the planar layout of a 384-well microplate. To fabricate EMSA units, we micro-molded/photo-polymerized a several-hundred-micron thick polyacrylamide gel (PAG)^35^,^38^.

Given our interest in advancing EMSAs to operate in a “screening mode”, we designed the EMSA card for simple operation such that the full array of 384 EMSA units is controlled with one slab-gel PAGE power supply and two electrodes. A series of 24 individual EMSA units create an electrical circuit between the single anode and the single cathode. The series configuration is repeated in parallel 16 times to yield the 384 unique – but concurrently actuated – EMSA units.

As each 24-plex series of EMSA units are fluidically and electrically connected, we next sought to minimize crosstalk (carry-over) among units of each series. We assessed the electrophoretic mobility of the ultra-high mobility fluorophore (AF647 dye, 0.02 mm^2^/Vs) to determine the longest acceptable EMSA duration (given the total electromigration distance) without crosstalk between two adjacent units. Electromigration of the high mobility standard under EMSA conditions (50 V/cm) established a maximum of 35 s for the EMSA duration over the 3.5-mm separation length for the eGFP-Fab EMSAs on the 384-plex EMSA card.

Originally developed for accurate, precise transfer of aqueous samples to microwell plates, the ADE technology can deliver 25 nL to 5 μL of sample to locations with <5% CV in volume precision^40^. Here, we interfaced the ADE liquid handling instrument with the EMSA card for Fab screening. For rapid alignment of the ADE fluid source with the *fs*PAG sample reservoirs, we developed a two-step registration process consisting of: (i) a printed target grid and (ii) a stack comprised of the source plate, the printed target grid, and the EMSA card. After registration of the sample reservoirs on the EMSA card to the target grid and source place, the ADE liquid handler dispensed to 384 sample reservoirs in 3 min, with an average droplet-center-to-reservoir-center displacement of <10% of the sample reservoir dimension in both horizontal and vertical directions (Figure S2). Sample dispensing to the 384-plex EMSA card was 85% faster than manual pipetting.

**Figure 3:**
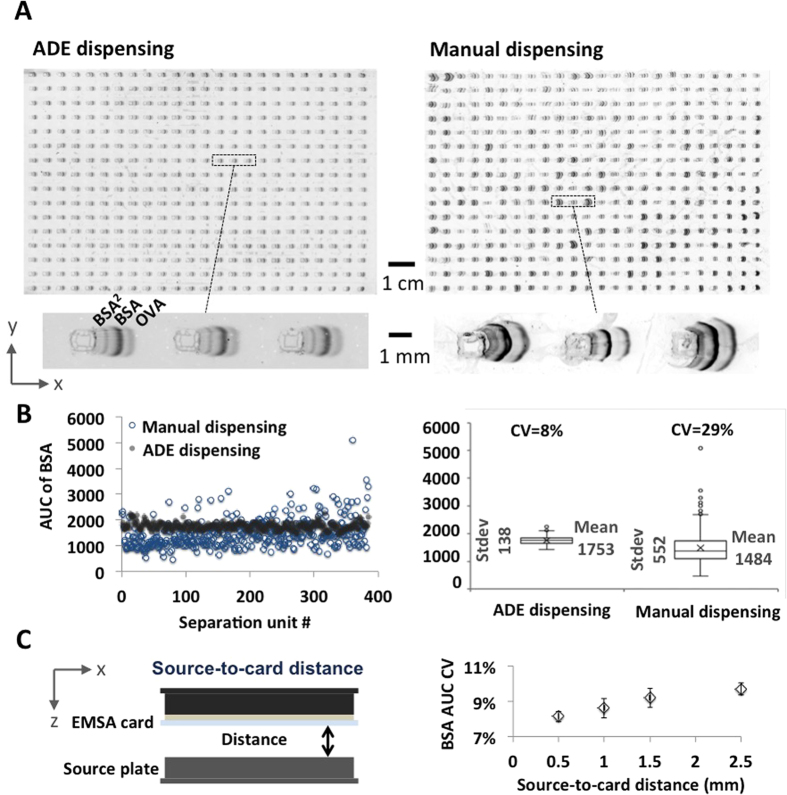
Technical variation in EMSA performance is reduced by ADE sample dispensing as compared to manual dispensing. (**A**) Fluorescence micrograph of PAGE of well-characterized protein ladder establishes quantitative estimates of technical noise in AUC and target mobility. Inset micrograph reveals the separation of protein ladder, including a BSA dimer (BSA^2^). Elapsed separation time 75 s; E = 50 V/cm. (**B**) Unit-to-unit variation in the AUC for the model BSA protein peak from the *fs*PAG assays displayed in (**A**). Left: A plot of BSA AUC value of both manual and ADE dispensing for *fs*PAG. Right: Box plot of BSA AUC value for both dispensing methods and their variation. (**C**) CV of the model BSA protein peak as a function of sample dispensing conditions (source-to-card distance). PAGE conditions are the same as in (**A**).

Next, we evaluated electrophoresis performance for both ADE dispensing and manual sample dispensing to the EMSA card. Here, we employed a well-characterized protein ladder (bovine serum albumin, BSA, at 66.5 kDa; ovalbumin, OVA, at 45 kDa; Fig. 3A). As is directly related to quantifying the K_D_, we measured how the AUC of BSA varies from unit-to-unit across each EMSA card. Technical variability in AUC directly affects the accuracy of EMSA K_D_ measurements, as discussed in the previous section. In intra-card AUC variability, with manual dispensing we observed CV = 29% (n = 384 units). In comparison, with ADE dispensing the CV measured in the AUC was 8% (n = 384 units, Fig. 3B).

As a corollary, we sought to understand the sensitivity of AUC variability to the distance between the ADE sample source plate and the EMSA card (source-to-card distance). A recent study has shown that^41^ stochastic instabilities in the droplet trajectory can occur, possibly due to variable ADE energy requirements at the surface of the source liquid surface. And minimizing the distance between the source and destination reduces the CV in droplet placement due to the reduced time during which the error in trajectory can propagate, thus improving the precision of the droplet placement. Increased positional accuracy of a droplet in the EMSA card reservoir would reduce dispensed-volume variation and would further promote droplet merging in the reservoir. We observed a modest decrease in CV with decreasing source-to-card distance, and the lowest CV in AUC was observed with a source-to-card distance of 500 μm (Fig. 3C).

We next sought to assess card-to-card variation in electrophoresis performance using the same AUC variability metric for the BSA ladder protein. In card-to-card variation, with manual dispensing we observed an AUC CV of 25% (n = 3 cards, each housing 384 separations) and with ADE dispensing an AUC CV of 3.6% (n = 3 cards, each housing 384 separations). Common EMSA-degrading performance modes observed in manual sample dispensing include both under-filled and over-filled sample reservoirs (Fig. 3A). In the case of initially over-filled sample reservoirs, EMSA separations exhibit notable transverse sample dispersion (“skew”). Protein peak skew arises from the protein molecules that lie outside the sample reservoir diffusing into the gel (in the direction perpendicular to the gel surface) prior to the initiation of electrophoresis, thus, locating these molecules in regions of slower electrophoretic mobility and non-uniform electric field. Along the separation axis, inconsistent sample volumes lead to variation in injection dispersion and, thus, variable peak widths and AUC. Taken together, in both unit-to-unit and card-to-card EMSA performance, we saw technical variation reduced appreciably with ADE sample dispensing onto the 384-plex EMSA cards.

Given the reduced intra-card and card-to-card EMSA variation with ADE sample dispensing, we next sought to identify unit-to-unit variation in EMSA performance that might arise from the geometric layout of the EMSA card. Here, we conjectured that even the 3 min ADE dispensing period required to fill all 384 reservoirs may lead to differential evaporative losses from the sample reservoirs loaded first versus reservoirs loaded at the end of the dispensing cycle. Evaporation of sample from the reservoirs would impact both AUC (volume changes) and electrophoretic mobility determination (conductivity changes). To assess any evaporative effects on EMSA performance, we applied an ANOVA test to scrutinize differences in peak AUC and migration distance of BSA between column 1 (first filled column) and column 24 (last filled column). At α = 0.05 the F_critical_ = 4, thus F >4 indicates significant variation between the two regions. In this comparison, ANOVA reported an F = 0.25 for AUC and F = 2.30 for migration distance, which suggested no significant spatial differences in sample concentration or electric conductivity between the sample reservoirs filled first (column 1) and those filled just before EMSA initiation (column 24).

Next, we evaluated separations performance between EMSA units near the perimeter of the EMSA card and EMSA units located in the interior of the device. We conjectured that the applied electric field variation (owing to electrode or device edge effects) might translate into EMSA performance variability. We compared AUC intensities and migration distance for BSA from an “interior region” of the EMSA card (defined as rows 2–15 and columns 2–23) to EMSA units along the perimeter of the device (rows 1 and 16, all columns; columns 1 and 24, all rows). In this analysis the F_critical_ = 3.84 at α = 0.05, and no perimeter EMSA units were observed to show significant performance differences from the interior region on both BSA AUC (row 1: F = 3.24; row 16: F = 3.47; column 1: F = 2.02; column 24: F = 0.76), and BSA migration distance (row 1: F = 2.74; row 16: F = 0.57; column 1: F = 0.20; column 24: F = 1.96). These results indicated no significant electrophoresis performance variation between units in the card interior and on the perimeter (Figure S3), which agrees with our findings on smaller 96-plex cards^38^.

### EMSA for K_D_ determination of recombinant antibodies

**Figure 4:**
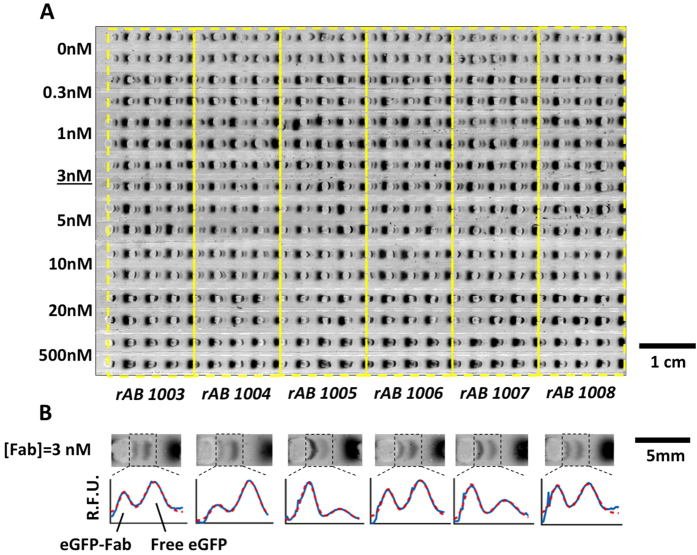
Screening-mode EMSAs of a six-member Fab library. (**A**) Fluorescence micrograph shows EMSA device with replicate units highlighted. Six Fabs (rAB 1003, rAB 1004, rAB 1005, rAB 1006, rAB 1007 and rAB 1008) were assayed simultaneously. Fabs were arranged column-wise with yellow boxed regions indicating EMSA card location of each of the six library members. (**B**) Fluorescence micrographs and intensity profiles across the six Fabs ([eGFP] = 0.3 nM, [Fab] = 0–500 nM, E = 50 V/cm, separation time = 28 s). R.F.U.: Relative fluorescence units.

**Figure 5:**
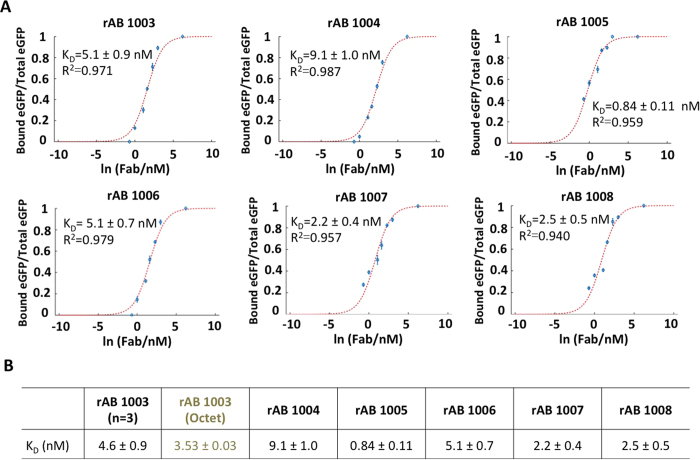
EMSA card reports the K_D_ of a six-member recombinant Fab library in 1 hour. (**A**) Dose-response curves acquired during EMSAs over a titration range of [Fab] with [eGFP] = 0.3 nM. Determination of the K_D_ values uses least squares fitting to 8-point concentration response with 8 replicates each. (**B**) Empirically determined K_D_ values for the six-member library (n = 3 for rAB 1003 for benchmark study), including comparison to the biolayer interferometry gold-standard (Octet) for rAB 1003. Sample and EMSAs conditions as in Fig. 4.

After validation, we applied the high-throughput ADE dispensing and EMSA card workflow to determine the K_D_ of six unique Fab fragments reactive to eGFP (Fig. 4A; Magnified fluorescence images and electropherograms of all Fab at 3 nM were displayed in Fig. 4B). We maintained a fixed eGFP concentration (0.3 nM) over a range of Fab concentrations and determined the K_D_ by least-squares regression^42^ to Equation (2). In 1 hour, the automated workflow acquired eight-replicates at each of eight-concentration points (0 <[Fab] <500 nM) for all six recombinant Fab fragments (rAB 1003, rAB 1004, rAB 1005, rAB 1006, rAB 1007 and rAB 1008; Fig. 5A). The K_D_ values are summarized compared to biolayer interferometry (Octet^®^) as is routinely utilized within the UCSF RAN pipeline (Fig. 5B).

In determining each K_D_, we sought to understand the variation in performance across each EMSA units. In considering all EMSAs with detectable eGFP-Fab immunocomplex at an elapsed separation time of 28 s (*E* = 50 V/cm), we observed *SR* = 0.968 ± 0.092 (n = 224, for units with resolvable peaks). The eGFP-Fab immunocomplex peak had an apparent electrophoretic mobility of (2.86 ± 0.26) × 10^−3^ mm^2^/Vs (n = 224). We further scrutinized the CV in AUC of the eGFP-Fab immunocomplex at different concentrations for each Fab in units with a detectable immunocomplex band (Figure S4). For all conditions measured, the immunocomplex AUC CV ranged from 5–20%, with lower CVs observed at higher [Fab] as expected, given that peaks with higher AUC are more readily resolved from neighboring (similar AUC) peaks. The highest CV of 20.8% was observed for the 1 nM rAB 1007 concentration (lowest concentration) and a tight CV of 5.9% was observed with the 10 nM concentration of rAB 1005.

To validate the K_D_ determined by the EMSA card, we quantified the K_D_ of eGFP binding with the Fab fragment rAB 1003 using biolayer interferometry (BLI), a component of the UCSF RAN workflow (Octet^®^ Red384 assay) and considered a gold-standard for molecular binding measurements. Octet is a surface-based binding kinetic assay^19^ that calculates K_D_ from direct measurements of the kinetic rate constants *k*_on_ and *k*_off_. Important to the Fab screening goals of this study is the copious consumption of target sample required by the instrument, which can present a challenge when determining K_D_ values for a library of recombinant antibodies. In light of limited sample volume availability, the Octet instrument was applied to one of the recombinant Fab molecules (rAB 1003, Figure S1). We determined K_D_ = 3.53 ± 0.03 nM as compared to K_D_ = 4.6 ± 0.5 nM (n = 3; triplicates for benchmarking) using the ADE and EMSA card workflow. While the K_D_ values agree within ~20%, we note that the Octet system is a heterogeneous assay (Fab immobilized to a solid phase) while the EMSA card is a homogeneous assay (all components are in solution phase). As the K_D_ value is known to be affected by surface immobilization of binding reagents^25^,^46^,^47^, making homogeneous measurements such as those reported by the EMSA card is an important complementary characterization to standard surface binding assays – especially when considering biological systems having solution-phase binding reactions.

## Discussion

To introduce a high-throughput, solution-phase system for determining the K_D_ of recombinant antibody libraries, we demonstrated, characterized, and applied an automated microfluidic sample dispensing instrument integrated with a highly multiplexed EMSA card. The integrated workflow harnessed 384 concurrent microfluidic EMSAs to quantify the K_D_ values of 6 recombinant antibody fragments generated for eGFP. The workflow reduced sample mass consumption by ~25-fold and improved the analytical throughput by ~5-fold, when benchmarked against commercial heterogeneous K_D_ characterization tools (Octet Red384). In mass consumption, EMSA-based K_D_ determination required ~0.1 μg of each Fab library member. In volume consumptions, the needed mass translated into a total 5 μL consumed of each Fab fragment and <1 μL consumed of the binding target (eGFP), both at a concentration of 10^−7^ M. Card fabrication, sample dispensing, and collection of 384 data points required 1 hour (i.e., 5 min for *fs*PAG fabrication, 20 min sample preparation, 3 min for sample dispensing on *fs*PAG with ADE, 1 min for EMSA run, 5 min for gel drying and 25 min for fluorescence imaging of the EMSA card).

In light of the expedited throughput, minimal sample consumption, and overall quantitative capacity of the microfluidic screening workflow, we are further maturing ADE-assisted EMSA cards for concurrent determination of K_D_ values for 16 recombinant Fab library members (8 concentration points with triplicate data, as a standard K_D_ measurement protocol), giving an analytical throughput of 16 K_D_ measurements/hour. Looking beyond screening of recombinant Fab libraries against target antigen, we are extending the *fs*PAG platform to binding pairs spanning a wide range of affinities and kinetic binding rates^48^ and, concomitantly, to high-sensitivity label-free detection. Further development and design innovation may allow simultaneous high-throughput characterization of the kinetic rate constants (*k*_on_ and *k*_off_), perhaps using modulation of geometry and photo-active materials in related mesofluidic card systems. Further, beyond the suitability of the *fs*PAG electrophoresis platform to K_D_ determination for recombinant antibody libraries, the ‘open format’ devices have proven suitable for determination of riboswitch conformation change upon binding to metabolites^38^ as well as quantifying the kinetic rate constants of binding reactions (*k*_on_, *k*_off_)^48^. Consequently, we view the *fs*PAG tool as applicable to a wide range of kinetic binding regimes, equilibrium conditions, and molecular masses (i.e., metabolites to antibodies). Nevertheless, the detection limits of the utilized fluorescence system constrain the measurable K_D_ to the nM range, thus necessitating development of high-sensitivity – and label-free – detection systems compatible with the *fs*PAG format.

Looking forward, we envision this study as forming the basis for streamlined affinity measurement tools to accelerate characterization and validation of recombinant antibodies, which may emerge as one way to standardize antibody production. The mesofluidic workflow is a quantitative assay that should aid immunoreagent developers at the bench for quickly assessing recombinant Fab reagents, assessing the impact of target antigen modification (i.e., conjugation with fluorophores, biotin, and other tags, and GFP-fusion proteins), as well as a wide variety of emerging alternative binding reagents (i.e., nanobodies, aptamers, riboswitches). The mesofluidic fsPAG card may also hold potential for disease diagnostic assays, where robust performance in complex matrix backgrounds is required, as has been studied for oral fluids^49^, mucosal and cerebral spinal fluids^50^, tear film fluids^51^, and blood components^52^,^53^,^54^ in molecular sieving matrix underpinning EMSA card performance.

## Methods

### Reagents

6 recombinant anti-eGFP Fab fragments (rAB 1003, rAB 1004, rAB 1005, rAB 1006, rAB 1007and rAB 1008) and eGFP were generated at UCSF Recombinant Antibody Network (RAN) according to protocols descried by Hornsby *et al*.^43^. eGFP was fluorescently labeled and purified in-house with an Alexa Fluor 647 (AF647) labeling kit (A20186, Thermo Fisher Scientific, Waltham, MA). After purification, residual free AF647 dye remained in the sample, as is common with purification of fluorophore conjugated proteins and useful for creating an internal electromigration control. Tween-20 and bovine serum albumin (BSA) powder were purchased from Sigma-Aldrich (St. Louis, MO). 4-(2-hydroxyethyl)-1-piperazineethanesulfonic acid (HEPES) buffer was purchased from Thermo Fisher Scientific. 10X tris-glycine buffer was purchased from Sigma Aldrich (St. Louis, MO). Food dye was purchased from McCormick (Sparks, MD). The protein ladder used in the EMSA card performance variability study include Alexa Fluor 555 (AF555) labeled Ovalbumin (OVA) and AF555 labeled Bovine serum albumin (BSA), both purchased from Thermo Fisher Scientific (Waltham, WA). Sample buffer used for delivery location accuracy study was made by diluting 10X tris-glycine buffer in water to a final concentration of 1X, and doped with red food dye.

### *fs*PAG fabrication

The *fs*PAG features and resultant EMSA card was fabricated through micro-molding. To create the micro-mold, Kapton^®^ tape (Kapton) of a given thickness was attached to a 12-inch silicon wafer and patterned with pre-defined geometry using a laser cutter (Full Spectrum laser model HL40-5G-110, Las Vegas, NV). The height of micro-mold was modulated by attaching multiple layers of Kapton up to the desired thickness. With the micro-molding, we fabricated 10%T, 3.3%C *fs*PAG structures using photopatterning. Briefly, the PAG precursor solution was prepared by mixing 10%T acrylamide (w/v), 3.3%C bis-acrylamide crosslinker (w/w) and 1% VA-086 photo-initiator (w/v) and degassed for 2–3 min under house vacuum with sonication and carefully poured over the micro-mold on silicon wafer. A surface-functionalized polymer sheet (Gelbond^®^, Lonza, Basel, Switzerland) was capped over the precursor solution and pressed against the surface of the micro-mold. The Gelbond^®^ substrate was tilted 4–5 times to ensure no bubbles were trapped in the mold. Excess precursor solution was removed with Kimwipe. Next, the silicon wafer was flipped and placed in the UV (ultra violet) exposure system (OAI model 30 collimated UV light source, San Jose, CA) with Gelbond^®^ facing downwards to the lamp for UV photo-polymerization. From empirical optimization, a 40 s exposure at 20 mW/cm^2^ was used to complete *fs*PAG formation. The EMSA card was ready for use immediately after UV polymerization. Otherwise, the EMSA card can be stored in water or buffer for long-term use. For the location accuracy and electrophoretic uniformity study, a 100 μm thick gel was used. For K_D_ measurements, a 400 μm thick gel was used.

### EMSA card and ADE integration

The Labcyte Echo^®^ 525 liquid handler (Echo) was used to perform the ADE sample transfer to the sample reservoirs on the EMSA cards. The EMSA card was aligned above a 384-well destination plate according to the “2-step registration” workflow described in the *SI*. In the performance variability studies, a protein ladder solution was dispensed into a 6-reservoir source plate (Labcyte Inc.) and sample transfer volume was set to 100 nL. For K_D_ measurements, solutions comprised of different concentrations of Fab and eGFP were placed in the 384-well source plate and the sample transfer volume was set to 400 nL. The automated sample dispensing step was performed with the Echo Plate Reformat software (Labcyte Inc.).

### Optimization of EMSA performance

To optimize the EMSA performance in a single-unit *fs*PAG structure, we prepared a binding reaction of 10 nM eGFP with 5 nM rAB 1003. The reaction was equilibrated prior to manually pipetting the sample solution into the single-unit EMSA card sample reservoir. A separation electric field of 50 V/cm was applied for 70 s.

### K_D_ measurements with EMSA card

Fab fragments and eGFP binding reactions were prepared in solutions containing 20 mM HEPES, 0.05% Tween-20 and 0.2% BSA (pH = 7.4). To measure the K_D_ value, solutions of 0.3 nM AF647-labeled eGFP were incubated in the buffer with various concentrations of Fab. The binding reaction was incubated for 2 hours before dispensing into the ADE tool and dispensed to the EMSA card. After sample dispensing to the sample reservoirs, the EMSA card was placed in a custom electrophoresis chamber^38^. To electrically address the device, two electrode wicks were sandwiched between the EMSA card and graphite electrodes placed on opposite ends of the gel lanes. Electrophoresis was performed at E = 50 V/cm for 28 s. Reactions of different Fab fragments were arranged column-wise with increasing concentrations in the vertical direction (Fig. 2B).

### Data acquisition and processing

Upon electrophoresis completion, the EMSA cards were removed from the chamber and dried in a nitrogen airflow for 5 min. Fluorescence imaging used an inverted epi-fluorescence microscope (Olympus IX-70) equipped with a 2X objective (PlanApo, N.A. = 0.08, Olympus, Center Valley, PA). Illumination light was sourced from an X-Cite^®^ exacte mercury lamp (Lumen Dynamics, Mississauga, Canada) with images acquired using a Peltier cooled charge-coupled device (CCD) camera (CoolSNAP HQ2, Roper Scientific, Trenton, NJ). Large-area imaging for the 384-plex EMSA cards was performed with Scan Slide function on Metamorph (Molecular Devices, Sunnyvale, CA). Image processing and subsequent data analysis were conducted with ImageJ (NIH, Bethesda, MD) and Matlab (Natick, MA). Fluorescence signals were averaged over the transverse direction of each EMSA separation lane to generate fluorescence intensity profiles with peaks fitted to Gaussian distributions. The intensity (area-under-curve) ratio of bound eGFP to total eGFP at each Fab concentration was calculated and least-square fitting of the binding equation was applied on the data to extract the K_D_ value.

### K_D_ measurements with Octet Red384

The EMSA card K_D_ measurements were benchmarked against Octet Red384 measurements using a 96-well format. Briefly, Anti-Human Fab-CH1 sensors were loaded with 150 nM Fab for 3 min including one non-loaded sensor as a control for non-specific binding of eGFP to the sensor. The association of eGFP at concentrations of 100 nM, 50 nM, 25 nM, 12.5 nM, 0 nM (background control), and 100 nM (sensor control) was measured for 15 min, followed by a 30-min dissociation phase. The sensors were regenerated with 100 mM glycine pH 1.5 to repeat the measurements. The K_D_ of rAB 1003 with AF647 labeled eGFP in HEPES buffer was made with both the Octet and the EMSA card workflow.

## Additional Information

**How to cite this article**: Pan, Y. *et al*. Determination of equilibrium dissociation constants for recombinant antibodies by high-throughput affinity electrophoresis. *Sci. Rep.* **6**, 39774; doi: 10.1038/srep39774 (2016).

**Publisher's note:** Springer Nature remains neutral with regard to jurisdictional claims in published maps and institutional affiliations.

## Supplementary Material

Supplementary Information
