# Supplementary material for: Determination of equilibrium dissociation constants for recombinant antibodies by high-throughput affinity electrophoresis
Source: Sci Rep. 2016 Dec 23;6:39774. doi: 10.1038/srep39774 (PMC5180089; doi:10.1038/srep39774)
Supplement: Supplementary Information [file srep39774-s1.pdf]

Supplementary Information

Determination of equilibrium dissociation constants for recombinant antibodies by high-throughput affinity electrophoresis

Yuchen Pan[1], Eric K. Sackmann[2], Karolina Wypisniak[3], Michael Hornsby[3], Sammy S. Datwani[2], and Amy E. Herr[1,4,*]

[1] University of California, Berkeley – UCSF Graduate Program in Bioengineering, Berkeley, CA, USA 94720

[2] Labcyte Inc., 1190 Borregas Ave, Sunnyvale, CA, USA 94089

[3] Department of Pharmaceutical Chemistry, University of California, San Francisco, CA USA 94158

[4] Department of Bioengineering, University of California, Berkeley, CA USA 94720

* Corresponding Author, Email: aeh@berkeley.edu

Table of Contents:

K_D measurements of Alexa Fluor® 647 labeled eGFP – rAB1003 binding on Octet Red384……2

2-step registration workflow for alignment of EMSA card to destination microplate 4

ANOVA analysis of unit-to-unit variation …………..…………………….…………………… 8

AUC of eGFP-Fab complex at each titration concentration for all Fab ……………………… 13

K_D measurements of Alexa Fluor® 647 labeled eGFP – rAB 1003 binding on Octet Red384

To benchmark the K_D measurements on *fs*PAGE, we performed binding analysis of Alexa Fluor® 647 (AF647) labeled eGFP and rAB1003 in HEPES. The concentration of the reagents and experimental conditions used in measurements were detailed in the *Methods* section.

The results were shown in Figure S1A. The reaction, composed of both association and dissociation processes, was monitored in real-time through the thickness of bio-layer, representing the formation/decomposition of binding complex. k_{on} and k_{off} were extracted by fitting the 1:1 kinetic equation to the binding curves. K_D values, then, were calculated by the ratio of k_{on} and $k_{off.}$ The resulting K_D is 3.53 ± 0.03 nM for the AF647 labeled eGFP - rAB1003. Detailed kinetic and affinity data are shown in Figure S1B.

A

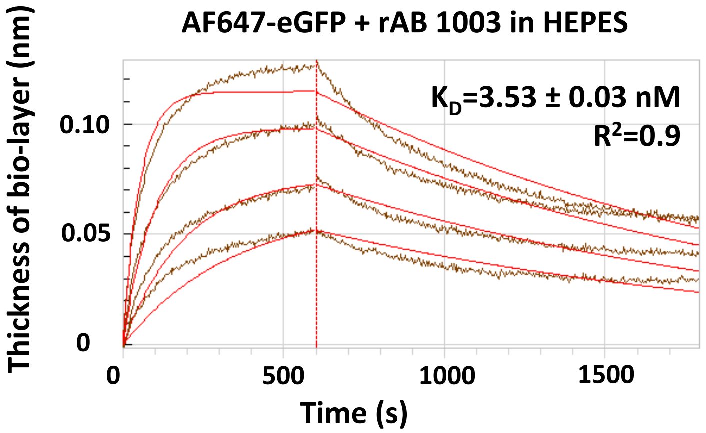

B

K_D (nM)	k_{on} ($M^{-1}S^{-1}$)	k_{off} (S^{-1})
3.53 ± 0.03	(10.3 ± 0.1) × 10^4	(7.43 ± 0.03) × 10^{-4}

Figure S1. K_D measurement of AF647 eGFP and rAB 1003 with Octet Red384. (A) Both raw kinetic data (brown) and fitting results (in red) were plotted. (B) Summary of kinetic and equilibrium data from experiments in (A).

2-step registration workflow for alignment of EMSA card to destination microplate

Registration proceeds in two steps: (i) printing of a target grid and (ii) assembly of a gel-grid-plate stack. (Figure S2A)

- First, a target grid outlining the geometry of 384 well plate was printed with ADE on a transparent microplate sealing film attached on the top surface of the destination plate, using a red food dye spiked water solution.
- In this method, the target grid is precisely mapped out on the location of where the droplet lands. Next the grids were labeled with permanent marker pen to register the location information for the next step.
- Next, the EMSA card was aligned onto the sealing firm with sample well overlapping the target grid.
- Finally, the gel-plate structure was inserted to Echo for liquid transfer.

Further, we set out to quantify the performance of the alignment and assess the location accuracy of the droplet dispensing. To do that, we integrated the ADE transfer with the 384-plex EMSA card and measured center-to-center displacement (Figure S2B) using a liquid sample doped with red dye. For quantitation of location accuracy, the bright-field image of a ADE loaded EMSA card was converted to grayscale and offset between the center of each droplet and the fluid well center was measured in both the horizontal and vertical directions (Figure S2C). In both dimensions, the concentration of the dye doped solution followed Gaussian distributions and least-square fitting was used to identify the droplet center position. The center position of each

fluid well was measured from a companion fluorescence image of each EMSA card, where (given the optically transparent nature of polyacrylamide gel) the fluid well boundaries are distinct and visible. Using this approach, we estimated that ADE dispensed droplets achieved an average horizontal displacement of 63.4 ± 52.5 μm (n = 384) and vertical displacement of 98.0 ± 75.3 μm (n = 384) when dispensed onto EMSA card (Figure S2D). Here, to minimize the impact of lateral diffusion of dye molecules on the quantification, we took the image bright-field image immediately after the transfer. With horizontal and vertical fluid well dimensions of 1 mm, respectively, the location accuracy along both axes was <10% of the respective fluid well dimension, indicating a high level of precision of gel-plate alignment with the "2-step registration" strategy.

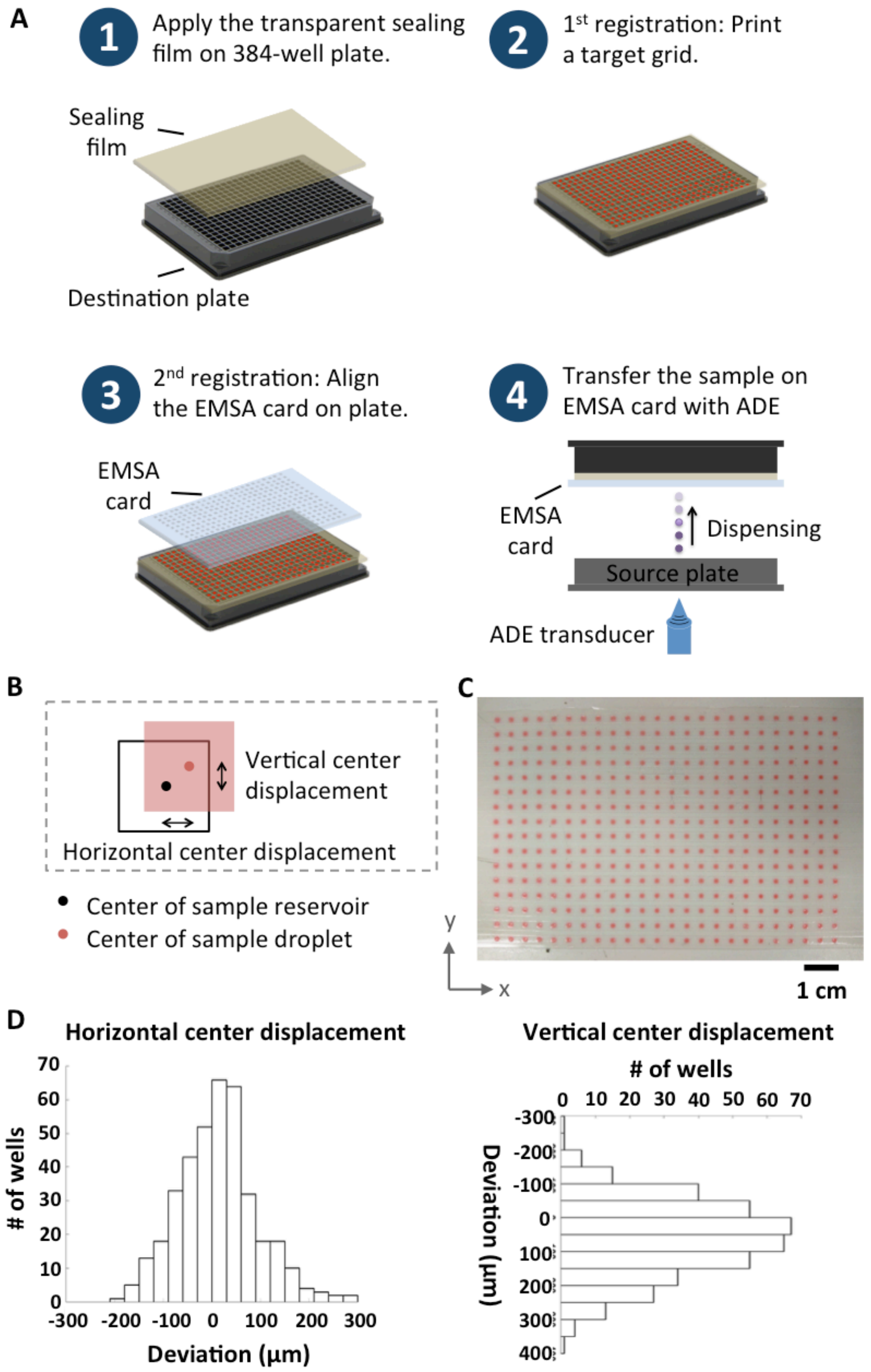

Figure S2. (A) 2-step registration workflow and location accuracy with ADE sample loading on *fs*PAG. (B) Schematics of horizontal and vertical center displacements. The displacements were

measured by taking the positional difference between centers of sample droplet and sample reservoir were recorded. (C) Brightfield image of ADE loaded 384-plex EMSA card. Sample liquid was doped with red dye. (D) Histogram of center displacement of liquid droplets and wells in both horizontal and vertical directions.

ANOVA analysis for unit-to-unit variation

The area-under-curve (AUC) and migration distance of BSA for unit-to-unit variation are listed in Figure S3.

A

1.73	1.53	1.63	1.71	1.87	1.53	1.76	1.64	1.85	1.71	1.87	1.71	1.86	1.71	1.59	1.49	1.57	1.77	1.76	1.81	1.65	1.51	1.71	1.53
1.61	1.66	1.58	1.73	1.59	1.68	1.75	1.49	1.58	1.49	1.56	1.78	1.59	1.65	1.83	1.69	1.73	1.77	1.96	1.64	1.49	1.57	1.64	1.58
1.52	1.39	1.51	1.56	1.77	1.75	1.63	1.46	1.69	1.55	1.64	1.57	1.62	1.65	1.71	1.79	1.88	1.93	1.81	1.62	1.54	1.54	1.46	1.65
1.68	1.51	1.63	1.65	1.51	1.82	1.79	1.54	1.61	1.67	1.48	1.64	1.55	1.53	1.63	1.69	1.67	1.71	1.73	1.61	1.57	1.46	1.48	1.61
1.53	1.49	1.56	1.53	1.55	1.69	1.67	1.74	1.81	1.64	1.60	1.61	1.55	1.58	1.67	1.75	1.80	1.85	1.70	1.51	1.46	1.43	1.54	1.52
1.64	1.49	1.47	1.54	1.58	1.60	1.71	1.68	1.64	1.90	1.69	1.53	1.47	1.71	1.74	1.76	1.55	1.62	1.63	1.50	1.47	1.44	1.41	1.61
1.42	1.49	1.59	1.63	1.55	1.68	1.58	1.75	1.72	1.75	1.50	1.61	1.38	1.64	1.67	1.60	1.57	1.81	1.72	1.55	1.55	1.39	1.46	1.52
1.57	1.61	1.80	1.79	1.80	1.70	1.81	1.54	1.86	1.69	1.76	1.69	1.71	1.77	1.70	1.56	1.60	1.88	1.94	1.69	1.60	1.59	1.73	1.65
1.52	1.43	1.73	1.81	1.79	1.64	1.67	1.77	1.73	1.57	1.57	1.56	1.47	1.45	1.63	1.59	1.72	1.92	1.64	1.55	1.50	1.42	1.43	1.53
1.65	1.55	1.51	1.63	1.60	1.61	1.73	1.82	1.55	1.74	1.73	1.64	1.53	1.76	1.70	1.65	1.75	1.77	1.58	1.58	1.80	1.79	1.46	1.64
1.57	1.54	1.61	1.55	1.58	1.81	1.95	1.80	1.87	1.54	1.56	1.44	1.55	1.44	1.66	1.63	1.66	1.77	1.71	1.60	1.59	1.40	1.49	1.48
1.62	1.67	1.38	1.56	1.59	1.69	1.58	1.59	1.61	1.79	1.92	1.59	1.55	1.75	1.80	1.58	1.54	1.64	1.52	1.52	1.68	1.56	1.58	1.60
1.67	1.65	1.67	1.63	1.65	1.70	1.72	1.80	1.79	1.82	1.78	1.62	1.70	1.52	1.81	1.59	1.61	1.70	1.57	1.64	1.57	1.86	1.57	1.63
1.78	1.56	1.62	1.52	1.54	1.78	1.81	1.74	1.82	1.99	1.69	1.55	1.43	1.67	1.69	1.68	1.75	1.51	1.55	1.72	1.58	1.59	1.66	1.90
1.52	1.43	1.49	1.72	1.51	1.65	1.72	1.72	1.49	1.71	1.71	1.85	1.55	1.65	1.77	1.64	1.94	1.70	1.61	1.60	1.78	1.51	1.45	1.65
1.50	1.53	1.64	1.73	1.95	1.51	1.64	1.81	1.75	1.90	1.48	1.80	1.77	1.60	1.88	1.51	1.59	1.73	1.83	1.70	1.54	1.76	1.68	1.71

Unit: × 10^4 R.F.U.

B

0.77	0.82	0.77	0.94	0.80	0.76	0.79	0.80	0.83	0.81	0.79	0.89	0.75	0.73	0.80	0.90	0.73	0.75	0.68	0.77	0.83	0.68	0.74	0.83
0.81	0.73	0.70	0.73	0.78	0.86	0.87	0.89	0.90	0.87	0.87	0.84	0.78	0.83	0.83	0.82	0.82	0.74	0.62	0.65	0.68	0.65	0.69	0.79
0.76	0.68	0.70	0.75	0.87	0.86	0.88	0.89	0.89	0.89	0.86	0.85	0.93	0.88	0.86	0.86	0.84	0.82	0.81	0.69	0.70	0.65	0.69	0.73
0.76	0.76	0.78	0.85	0.90	0.88	0.86	0.86	0.87	0.87	0.88	0.82	0.83	0.76	0.84	0.93	0.80	0.84	0.80	0.79	0.85	0.68	0.70	0.80
0.76	0.73	0.80	0.84	0.86	0.85	0.81	0.86	0.88	0.86	0.85	0.86	0.82	0.86	0.82	0.85	0.83	0.81	0.82	0.76	0.68	0.76	0.65	0.80
0.77	0.80	0.84	0.80	0.86	0.85	0.83	0.86	0.93	0.95	0.86	0.87	0.88	0.85	0.78	0.76	0.78	0.79	0.79	0.74	0.70	0.65	0.69	0.78
0.79	0.82	0.79	0.78	0.78	0.79	0.77	0.77	0.85	0.83	0.80	0.84	0.81	0.82	0.77	0.76	0.80	0.80	0.77	0.74	0.69	0.72	0.75	0.77
0.89	0.82	0.79	0.82	0.82	0.78	0.77	0.77	0.81	0.83	0.79	0.82	0.75	0.79	0.79	0.77	0.79	0.78	0.79	0.76	0.76	0.78	0.77	0.85
0.88	0.88	0.91	0.91	0.89	0.76	0.83	0.82	0.82	0.82	0.76	0.78	0.78	0.87	0.89	0.77	0.78	0.82	0.79	0.78	0.78	0.76	0.74	0.73
0.86	0.90	0.88	0.91	0.87	0.82	0.84	0.80	0.78	0.85	0.80	0.81	0.80	0.85	0.87	0.95	0.82	0.81	0.82	0.78	0.81	0.79	0.73	0.73
0.89	0.88	0.88	0.90	0.86	0.78	0.76	0.74	0.77	0.82	0.79	0.82	0.80	0.83	0.85	0.84	0.83	0.79	0.78	0.78	0.88	0.79	0.72	0.72
0.86	0.84	0.85	0.83	0.81	0.80	0.79	0.77	0.78	0.77	0.78	0.88	0.83	0.84	0.88	0.89	0.87	0.82	0.80	0.77	0.77	0.80	0.75	0.86
0.86	0.86	0.84	0.82	0.79	0.81	0.76	0.79	0.81	0.78	0.79	0.83	0.81	0.79	0.86	0.90	0.94	0.89	0.87	0.84	0.83	0.84	0.78	0.81
0.83	0.88	0.86	0.84	0.76	0.75	0.82	0.78	0.79	0.78	0.83	0.85	0.90	0.88	0.87	0.87	0.84	0.79	0.77	0.74	0.80	0.80	0.78	0.74
0.83	0.77	0.78	0.96	0.78	0.78	0.74	0.86	0.79	0.78	0.85	0.83	0.82	0.83	0.84	0.86	0.85	0.82	0.81	0.74	0.78	0.79	0.79	0.82
0.75	0.84	0.80	0.77	0.81	0.81	0.77	0.95	0.75	0.81	0.80	0.88	0.77	0.76	0.78	0.73	0.73	0.72	0.72	0.73	0.76	0.96	0.95	0.88

Unit: mm

Figure S3. Matrix of BSA AUC and migration distance in 384-plex EMSA card. (A) BSA AUC values. R.F.U.: Relative fluorescence units. (B) BSA migration distance values.

First to test the spatial variation intrinsic to the *fs*PAGE assay, we actively sought for any side effects. The ANOVA tests were applied between interior group (defined as rows 2 -15 and columns 2 – 23) and each of the perimeter groups (row 1 and 16, all columns; column 1 and 24,

all rows) on the *fs*PAGE result. (Some values listed were rounded). At $\alpha = 0.05$ the $F_{critical} = 4$, thus $F > 4$ indicates significant variation between the two regions.

<u>For BSA AUC:</u>

ANOVA test between interior group and row 1:

The sum of squares (SS) of all units $SS_t = 5.02 \times 10^8$

The sum of SS in both group: $SS_w = 4.97 \times 10^8$

The SS between groups: $SS_b = SS_t - SS_w = 0.05 \times 10^8$

Degree of freedom (DOF) within groups: 330

DOF between groups: 1

Therefore the mean square (MS) within groups: $MS_w = 0.0488 \times 10^8$

MS between groups: $MS_b = 0.015 \times 10^8$

$F_{Row1} = MS_b / MS_w = 3.24$

Similarly for row 16:

$SS_t = 5.12 \times 10^8$, $SS_w = 5.07 \times 10^8$, $SS_b = 0.0533 \times 10^8$, $MS_w = 0.0533 \times 10^8$, $MS_b = 0.0154 \times 10^8$.

$F_{Row16} = 3.47$

For column 1:

SS_t= 4.80 × 10^8, SS_w= 4.77 × 10^8, SS_b= 0.029 × 10^8, MS_w= 0.029 × 10^8 , MS_b= 0.014 × 10^8. F_{Col1} =2.02

For column 24:

SS_t= 4.79 × 10^8, SS_w= 4.78 × 10^8, SS_b= 0.011 × 10^8, MS_w= 0.011 × 10^8 , MS_b= 0.014 × 10^8. F_{Col24} = 0.76.

<u>For BSA migration distance:</u>

ANOVA test between interior group and row 1:

The sum of squares (SS) of all units SS_t= 1.14 mm^2

The sum of SS in both group: SS_w=1.13 mm^2

The SS between groups: SS_b= SS_t-SS_w=0.01 mm^2

Degree of freedom (DOF) within groups: 330.

DOF between groups: 1

Therefore the mean square (MS) within groups: MS_w= 0.0094 mm^2

MS between groups: MS_b=0.0034 mm^2

F_{Row1}= MS_b/ MS_w=2.74

Similarly for row 16:

SS_t= 1.1676 mm^2, SS_w=1.1656 mm^2, SS_b=0.002 mm^2, MS_w=0.002 mm^2, MS_b=0.0035 mm^2. F_{Row16} =0.57

For column 1:

SS_t= 1.0815 mm^2, SS_w= 1.0809 mm^2, SS_b= 0.0007 mm^2, MS_w= 0.0007 mm^2, MS_b= 0.0033 mm^2. F_{Col1} =0.20

For column 24:

SS_t= 1.0859 mm^2, SS_w= 1.0795 mm^2, SS_b= 0.0064 mm^2, MS_w= 0.0064 mm^2, MS_b= 0.0033 mm^2. F_{Col24} =1.96.

All F value calculated between side perimeter groups and interior groups are below $F_{critical}$ = 4. Therefore, these results indicated no significant variation between the EMSA performance in units in the chip interior and on the perimeter (Figure S3), which also corroborated our analysis of low-density 96-plex *fs*PAG devices[36].

Next to seek any spatial variation due to the sample differential evaporation during the sample delivery stage, we applied an ANOVA (analysis of variance) test to scrutinize differences in AUC and migration distance of BSA between column 1 (first filled column) and column 24 (last filled column).

<u>For BSA AUCs:</u>

SS_t= 2.82 × 10^8, SS_w=2.79 × 10^8, SS_b=0.023 × 10^8, MS_w=0.093 × 10^8, MS_b=0.023 × 10^8. $F_{1\text{-}16p}$ =0.25

<u>For BSA migration distance</u>, similarly, the $F_{1\text{-}16m}$ =2.30

The analysis indicated negligible effects of AUC variation stemming from evaporation or ADE dispensing on the EMSA performance.

AUC of eGFP-Fab complex at each titration concentration for all Fab

The fitted area-under-curve (AUC) of eGFP-Fab complex bands for all Fabs at each Fab titration concentration are shown in the boxplots below.

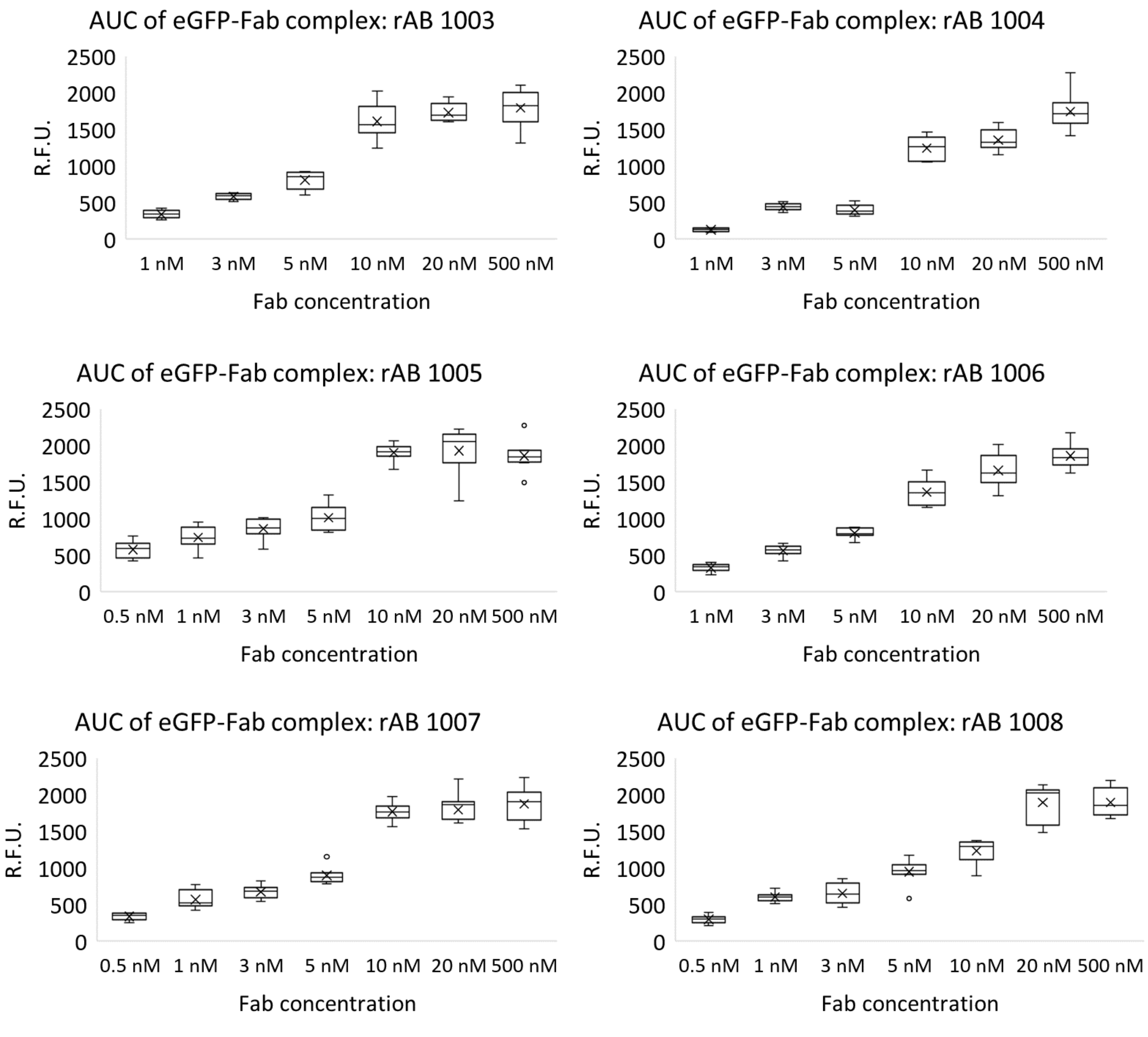

Figure S4. Boxplots of AUCs of eGFP-Fab complex band at difference Fab concentration (Six Fabs; rAB 1003, rAB 1004, rAB 1005, rAB 1006, rAB 1007, rAB 1008). R.F.U.: relative fluorescence units.
